# Feeding Intolerance among Preterm Neonates Admitted to the Neonatal Intensive Care Unit at a Tertiary Care Centre: A Descriptive Crosssectional Study

**DOI:** 10.31729/jnma.7826

**Published:** 2022-11-30

**Authors:** Pawana Kayastha, Sabina Shrestha, Ashish Subba

**Affiliations:** 1 Department of Paediatrics, Kathmandu Medical College, Sinamangal, Kathmandu, Nepal

**Keywords:** *feeding patterns*, *food intolerance*, *morbidity*, *mortality*, *preterm infants*

## Abstract

**Introduction:**

Premature infants frequently suffer from feeding intolerance related to prematurity and are highly associated with morbidity and mortality. Breast milk is fundamental to the improvement of the infant's immature vulnerable framework and decreases child mortality. The aim of this study was to find out the prevalence of feeding intolerance in premature neonates admitted to the neonatal intensive care unit of a tertiary care centre.

**Methods:**

This descriptive cross-sectional study was carried out among premature infants admitted to the Neonatal Intensive Care Unit of a tertiary care hospital from 15 December 2021 to 15 May 2022 after receiving ethical approval from the Institutional Review Committee (Reference number: 2211202103). Convenience sampling was done. Point estimate and 95% Confidence Interval were calculated.

**Results:**

Among the 55 preterm neonates, the prevalence of feeding intolerance was 21 (38.18%) (25.34-51.02, 95% Confidence Interval).

**Conclusions:**

The study showed that the prevalence of feeding intolerance among neonates was higher than in studies conducted in similar settings.

## INTRODUCTION

Feeding intolerance is defined as the inability to digest enteral feedings associated with increased gastric residuals, abdominal distension and/or emesis in neonates.^1^ Establishing enteral feeding is a critical milestone in premature infants.^2^

The main cause of neonatal deaths in Nepal is prematurity (30.8 %).^3^ Provision of breast milk is an effective way of preventing the 2.8 million annual infant deaths worldwide.^4^ A significant reduction in neonatal mortality rate (NMR) was observed in Mumbai and an increased survival rate for preterm with the establishment of a milk bank.^5^

The objective of this study was to find out the prevalence of feeding intolerance among preterm infants admitted to the neonatal intensive care unit (NICU) of a tertiary care hospital.

## METHODS

This descriptive cross-sectional study was carried out among all the cases with the diagnosis of prematurity admitted to the NICU of Kathmandu Medical College during a period of six months from 15 December 2022 to 15 May 2022 after receiving ethical approval from the Institutional Review Committee of KMC (Reference number: 2211202103). The study included all admitted preterm infants of both genders from day zero to day 28 of life and with gestational age (GA) from 22 to 36 weeks (estimated by 1^st^ day of the maternal last menstrual period). The study excluded any neonates suffering from intestinal congenital anomalies, neonates with fulminating sepsis from the onset, also neonates with milk allergies. Convenience sampling technique was done. The sample size was estimated using the formula:


$$\begin{array}{ll} \text{n} & ={\text{Z}}^{2}\times \frac{\text{p}\times \text{q}}{{\text{e}}^{\text{2}}}\\ & =1.96^{2}\times \frac{0.042\times 0.958}{0.05^{2}}\\ & =43 \end{array}$$

Where,

n= minimum required sample size,
Z= 1.96 at 95% Confidence Interval (CI),
p= prevalence of feeding intolerance among premature neonates taken from a previous study, 4.2%^2^
q= 1-p
e= margin of error, 5%

The calculated sample size was 43. A total of 55 preterms were included in this study.

Diagnosis of feeding intolerance is considered by the presence of one or more signs that lead to interruption of the enteral feeding regime of the preterm as increased gastric residuals (>50%) of the previous feeding, greenish residual, hemorrhagic residual, emesis, abdominal distention (increase in abdominal girth by two centimetres or more in between feedings), bloody stool, diarrhoea, visible bowel loops and apnea.^1^ All the studied neonates were subjected to full history taking and full clinical examination.

The collected data were organised, tabulated, and statistically analysed by using IBM SPSS Statistics version 20. Point estimate and 95% CI were calculated.

## RESULTS

Among 55 preterm neonates, the prevalence of feeding intolerance was seen in 21 (38.18%) (25.34-51.02, 95% CI). The gestational age of enrolled infants ranged from 24 to 36 with a mean of 32.09±3.01 weeks of gestation. Similarly, the mean birth weight ranged from 0.70 grams to 2.60 grams with a mean of 1.65±0.48 grams. The mean time of diagnosis of feeding intolerance was found 3.61±1.68 days with a range from one to eight days. Among 21 preterms with feeding intolerance 13 (61.90%) were born at 28-32 weeks and 8 (38.10%) were at 33-35 weeks of gestation. A total of 10 (47.62%) preterm infants had clinical features of abdominal distension, 5 (23.81%) had apnea, 3 (14.29%) had gastric residues, and 3 (14.29%) had vomiting (Table 1).

**Table 1:** Distribution of the study patients by signs of feeding intolerance (n= 21).

Signs of feeding intolerance	n (%)
Abdominal distension	10 (47.62)
Apnea	5 (23.81)
Gastric residues	3 (14.29)
Vomiting	3 (14.29)

In feeding intolerance 7 (33.33%) patients were found to have Hyaline Membrane Disease (HMD) followed by 4 (19.05%) with sepsis, and 3 (14.29%) with pneumonia (Table 2).

**Table 2:** Morbidities among patients with feeding intolerance (n= 21).

Morbidities	n (%)
Hyaline Membrane Disease (HMD)	7 (33.33)
Meconium Aspiration Syndrome (MAS)	1 (4.76)
Meningitis	2 (9.52)
Necrotizing Enterocolitis (NEC)	2 (9.52)
None	1 (4.76)
Pneumonia	3 (14.29)
Seizure	1 (4.76)
Sepsis	4 (19.05)

Most of the preterm infants, 48 (87.3%) were underexpressed breast milk and 7 (12.7%) were formula fed.

## DISCUSSION

In this study, it was observed that the gestational age of preterm neonates ranged from 28 to 36 with a mean of 32.09±3.01 weeks of gestation which was similar (32.1±2.3 weeks with a range from 28 to 36 weeks) to the study result done at a general hospital of Dhaka.^2^ A study done in Western Nepal shows similarity to this study in findings as the birth weight of babies ranged from 0.935 to 2.55 kg with a mean of 1.55 kg and this study also showed the birth weight of preterms ranged from 0.70 gram to 2.60 gram with mean of 1.65±0.48.6.

In this study, the mean time of feeding intolerance diagnosis was found 3.61±1.68 days with a range from one to eight days whereas time at feeding intolerance diagnosis ranged widely from 3 to 25 days in a study done at Egypt.^7^ The incidence of feeding intolerance also varied greatly in different studies done in different geographical areas, 4.2% in Dhaka, 36% in Bangladesh, 2.6% in Egypt, 34% in African Israelian and 15% in other Israelian, 11.02% in Western Nepal.^2,6-10^ The prevalence of feeding intolerance in this study was found to be 38.18% which is almost similar to a study done in Bangladesh,^8^ but higher than another Bangladeshi study.^2^ Here, the majority of preterm with feeding intolerance had features of, abdominal distension (47.6%) and some had apnea (23.8%), gastric residual 14.3%, and vomiting (14.3%). But unlike this study, in Western Nepal, 90.7% of all feed intolerant babies had vomiting followed by gastric residue, abdominal distension, reduced or absent bowel sounds and apnea respectively.^6^ Most of the other studies also had similar clinical presentations of vomiting, abdominal distension, gastric residual, and apnea.^2,7^ In this study, as regards the presence of other morbidities in the cases with feeding intolerance, it was HMD (33.3%) followed by sepsis (19%), and pneumonia (14.3%). Similar to ours, another study also detected HMD as the most common morbidity (23.7%), in preterm with feed intolerance.^6^

In our observation most preterm neonates admitted to NICU (87.27%) started with expressed breast milk and the remaining (0.12%) were given formula milk. The World Health Organisation recommends the use of donor human milk (DHM) as a lifesaving alternative, especially for vulnerable, low birth weight, and preterm neonates.^10^

This study was conducted in a single centre with a limited sample size and a short time period for data collection, because of which the results may not be generalised to all preterm neonates. Adverse maternal conditions were not considered as exclusion criteria. Also, FI is a clinical diagnosis with an equivocal definition, which can be confused with other diseases.

## CONCLUSIONS

The study concluded that the prevalence of feeding intolerance among neonates was higher than in other national and international studies conducted in similar settings. Identifying the prevalence and knowing feeding practices in premature neonates will help us reduce mortality and morbidity due to prematurity.
